# Supplementary material for: Amphibian (Xenopus laevis) Macrophage Subsets Vary in Their Responses to the Chytrid Fungus Batrachochytrium dendrobatidis
Source: J Fungi (Basel). 2025 Apr 15;11(4):311. doi: 10.3390/jof11040311 (PMC12028672; doi:10.3390/jof11040311)
Supplement: Supplementary file 1 [file jof-11-00311-s001.zip › jof-3560537-supplementary.pdf]

Supplementary Materials

Supplemental Table S1. List of primer sequences

Primer target	Sequence 5′→3′
X. laevis-specific gene targets	
arg1 FWD<br>*arg1* REV	TCCAAGGGACAGCCAAGAAG<br>CTCGAACATCATTGCCAAATTC
gapdh FWD<br>*gapdh* REV	ATGTGTCCGTTGTGGATCTG<br>GATTCCTTTCATTGGTCCCTCT
ido FWD<br>*ido* REV	TATTGCGGATGCGAGAGTACA<br>TCCCCGCTTTCTTGAACGTA
il10 FWD<br>*il10* REV	CAGTCCGTGTCTGAAACAATTC<br>CAGCAACTTGTCCTTGAGAAAG
inos FWD<br>*inos* REV	TTGGCCTGAGGTATACGT<br>CCCATGTACCAGCCGTTGA
noxa2 FWD<br>*noxa2* REV	CAAACAACCCTCCTTCTCGTCCAA<br>GGACCTGCATTTCTTCTGCTGTCT
nox2 FWD<br>*nox2* REV	TAACATCTACCTCACTGGCTGGGA<br>CCAGTTTGGTCTGCCATACAAGGT
tnf FWD<br>*tnf* REV	TGTCAGGCAGGAAAGAAGCA<br>CAGCAGAGCAAAGAGGATGGT
B. dendrobatidis-specific primers	
amd1 FWD<br>*amd1* REV	TGCAGGGCATCGTGTATT<br>TAGCAGGCCATTCAAAGAGTAG
arg FWD<br>*arg REV*	GGCTCAAACCCTGCTTTAAAC<br>GAAGGCCTTGATGCCATACT
cat FWD<br>*cat* REV	CTTGGCACACCTTGTCAAATATC<br>CATGGTTGAAGTTGGATTG
cda FWD<br>*cda* REV	CGCGTTTGGAACGTGTAAAT<br>AGCAGACAACTTGGGATCAG
gapdh FWD<br>*gapdh* REV	GTTCACTCTGTTACTGCTACCC<br>GTGGAGGCAGGAATGATGTT
ido FWD<br>*ido* REV	TCTCATACATTCACCGGAACAG<br>CCTGCTGGGCATCTACTAATC
its1 FWD<br>*its1* REV	GCCATATGTCACGAGTCGAA<br>GCCAAGAGATCCGTTGTCA
odc FWD<br>*odc* REV	CTCGATATTGGAGGTGGGTTTC<br>GTCTCGGGAAAGTTGTCATCA
sdm FWD<br>*sdm* REV	TACCATCATCTGCGTTAAA<br>TGCAGGACCTCATTGGAATC
srm FWD<br>*srm* REV	CTGAGCGAGATGAGTTCTCTTAC<br>CATCACCACCACCGATAACA

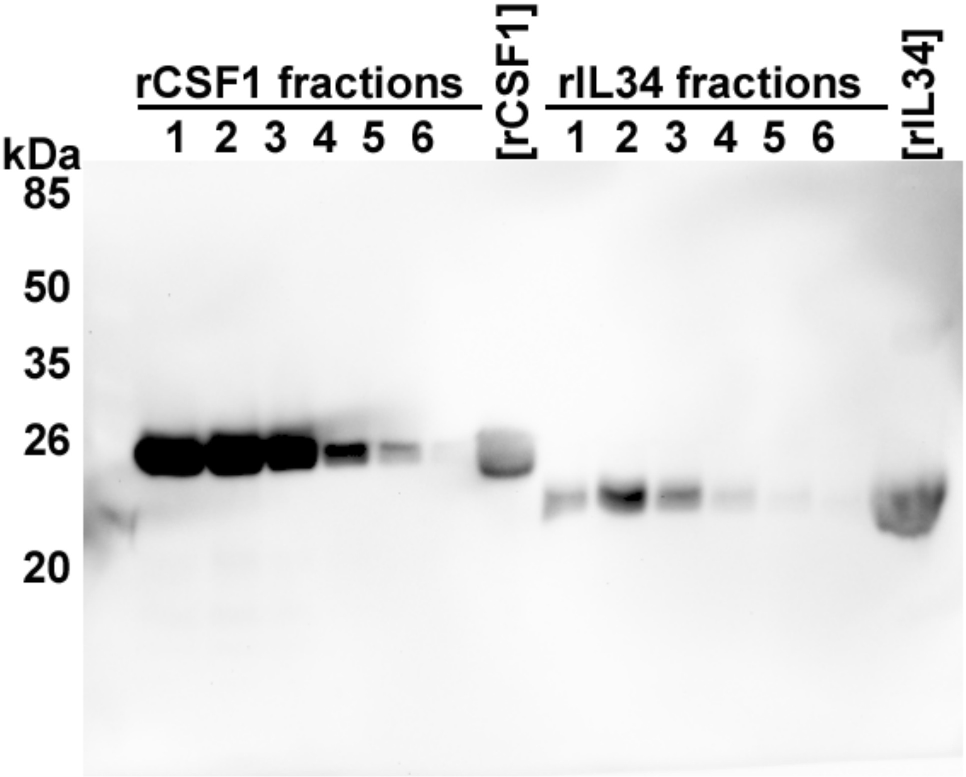

Supplemental Figure S1. Isolation of rCSF1 and rIL34. Recombinant (r)CSF-1 and rIL-34 were produced using an Sf9 insect cell expression system. The recombinant CSF-1 and IL-34 were eluted in fractions. The fractions containing the protein were pooled and concentrated, denoted as [rCSF1] and [rIL34]. Proteins were resolved by SDS-gels and western blot against the V5 epitope.
